# Association between low estrogen receptor positive breast cancer and staining performance

**DOI:** 10.1038/s41523-020-0146-2

**Published:** 2020-02-05

**Authors:** Dennis Caruana, Wei Wei, Sandra Martinez-Morilla, David L. Rimm, Emily S. Reisenbichler

**Affiliations:** 1 Department of Pathology, Yale University School of Medicine, New Haven, CT USA; 2 Yale University School of Public Health, New Haven, CT USA; 3 Department of Oncology, Yale University School of Medicine, New Haven, CT USA

**Keywords:** Tumour biomarkers, Predictive markers, Breast cancer

## Abstract

Estrogen receptor (ER) expression in breast carcinomas, determined by immunohistochemistry, indicates statistically significant benefit to endocrine therapy in patients with tumors expressing ER in ≥1% of tumor cells. Rare cases with low ER expression (1–10%) lead to the dilemma of treating these tumors as ER positive or negative. We hypothesize that low ER positive result from poor staining performance and that we may detect this artefact by assessing the average dynamic range of normal ducts adjacent to low ER positive tumors. Using quantitative tools, we compare the dynamic range of normal background ER expression in patients with low (1–10%) ER tumors to dynamic range of ER expression in normal epithelium from control patient populations, to determine if low ER cases are accompanied by decreased dynamic range. Low ER cases were infrequent (1% of invasive breast carcinomas). Twenty-one cases with low ER staining and two control cohorts, including a tissue microarray (TMA) of 10 benign breast sections and a group of 34 control breast carcinomas (reported as ER negative or >10% ER positive) with normal background epithelium, were digitally scanned. QuPath was utilized to quantify ER staining for each cell as the mean optical density of nuclear DAB staining. The dynamic range of ER expression in normal epithelium surrounding low ER tumors was significantly lower (range 2–240, median 16.5) than that of the benign epithelium in the control tumors (range 3–475, median 30.8; *p* < 0.001) and benign TMA sections (range 38–212, median 114; *p* < 0.001) suggesting inconsistent stainer performance.

## Introduction

Estrogen receptor (ER) is expressed in the majority of invasive breast carcinomas and is an important predictive and prognostic marker. Many variables in tissue processing and testing can affect the level of ER expression seen in breast tumors.^1–5^ For this reason, all invasive and recurrent breast carcinomas are tested for ER expression by immunohistochemistry (IHC) in accordance with guidelines regarding specimen handling, laboratory testing and interpretation of the results.^6^ In spite of the broad dynamic range of ER expression in both tumor and normal breast epithelium, ER expression in tumors is reported as a final dichotomized, positive or negative result. Specifically, the standard of care is to report the percentage of cells positive “at any intensity” but there is no routine method for standardization for intensity. Instead, we depend on standardized, often closed system staining protocols to define the intensity threshold. While this method appears to have been effective in standardization, if the threshold moves up or down, the percentage of positive cells could also vary, but in a manner that goes undetected. In fact, years ago, this approach led to some significant under-calling of ER positive cases in some provinces in Canada.^7,8^

Based on data showing that tumors with at least 1% ER positive nuclear staining at any intensity receive a statistically significant benefit to endocrine therapy,^9^ the American Society of Clinical Oncology/College of American Pathologists (ASCO/CAP) published guidelines in 2010 recommending that any tumor showing ≥1% ER expression be reported as a “positive” result.^6^ However, Raghav et al later found that patients with tumors expressing 1–5% ER receive no clinical benefit from endocrine therapy.^10^ Most invasive tumors however, have been shown to demonstrate a binary distribution of ER expression, being either diffusely positive or negative.^11–13^ However, a small percentage of cases show low level ER expression, defined as nuclear staining of any intensity in 1–10% of tumor cells. This finding may lead to dilemmas in treatment decisions for these patients. When this reading occurs in tumors that are also progesterone receptor (PR) and human epidermal growth factor receptor 2 (HER2) negative, recategorization as triple negative tumors is often considered. If these tumors are truly triple negative tumors, a low ER expression level could erroneously exclude patients from newer poly (ADP-ribose) polymerase (PARP) inhibitor related therapies or clinical trial eligibility. Conversely, if the low-level staining is real, then withholding endocrine therapy in these cases could result in undertreatment of a potentially responsive tumor.

Staining of hormone receptors is highly regulated in an attempt to ensure equal testing regardless of testing location, but low levels of ER expression are not always reproducible. Although methods have been published for standardization of the ER threshold,^14^ these methods have not seen commercial success. Thus, most individual labs do not have the capacity to clinically validate the specific ER positive cutoffs for each laboratory that correspond to clinical benefit to endocrine therapy. Studies have shown a lack of reproducibility between pathologists when manually interpreting ER expression at very low levels.^15^ The 2010 ASCO/CAP guidelines recognize this potential difficulty and state that “it is reasonable for oncologists to discuss the pros and cons of endocrine therapy with patients whose tumor contain low levels of ER by IHC…and to make an informed decision based on the balance”.^6^ When no or little ER staining is seen in tumor cells, a crucial quality control element is to ensure adequate fixation and staining occurred by evaluation of external, and preferably, internal control tissue. Although the current guidelines state that any internal control epithelium should demonstrate heterogeneous staining in luminal epithelial cells, this quality control element is often reported as positive, negative or absent internal control tissue present, without recognition of the expected dynamic range of biologic expression seen in normal breast ducts. Normal luminal breast epithelium should demonstrate a broad range of ER expression by IHC which may be affected by both physiologic and laboratory variables.^16^ While the human eye may detect the presence or absence of variable staining intensity, image analysis allows accurate quantification of the variation in individual cells^17^ and potentially allows the dynamic range of expression in these ducts to be used as an internal standardization factor.

The dynamic range of expression is defined as the difference in intensity between the lowest detectable level and the highest level. This range would be expected to be large, reflecting the biological difference within a duct, of cells expressing very few ER molecules compared to those expressing thousands of molecules. If there is an issue with the staining process, or any pre-analytic variable that artifactually decreases the sensitivity of the test, this might manifest itself in a decrease in the dynamic range measured in normal ducts. We hypothesize that low ER positive cases may sometimes be a result of poor staining performance and that we may be able to detect this artefact by assessing the average dynamic range of normal ducts adjacent to low ER positive tumors. Here, using quantitative tools, we compare the dynamic range of normal ER expression in patients with low ER tumors to the dynamic range of ER expression in normal epithelium from control patient populations, to determine if low ER cases are sometimes accompanied by decreased dynamic range, suggesting that the low level of expression is an artefact of laboratory or stainer specific issues.

## Results

### Low ER carcinoma clinical cases

**Fig. 1:**
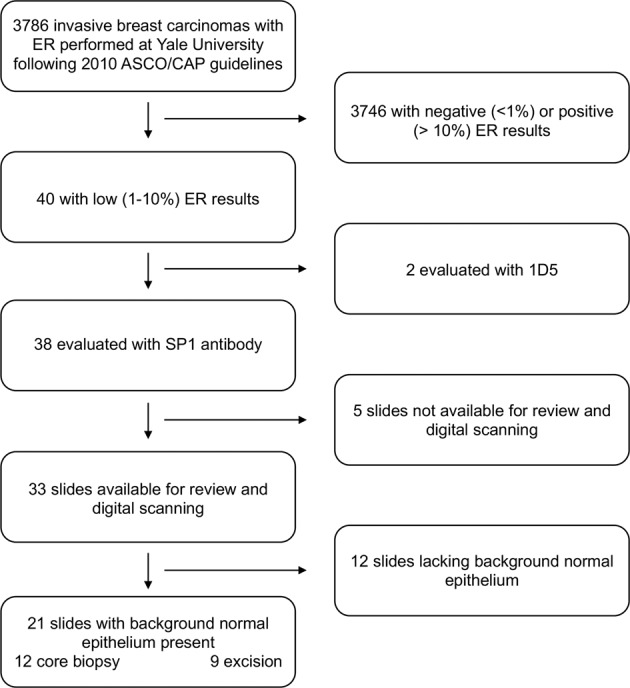
Consort diagram of clinical case selection.

The pathology database search identified that ER IHC was performed on 3786 cases of invasive breast carcinomas following the updated 2010 ASCO/CAP guidelines, 40 (1.05%) of which were reported as demonstrating low (1–10%) ER expression. Following exclusion of cases stained with the 1D5 antibody, review of the available stained sections revealed normal background epithelium in 21 cases, 12 of which were core biopsy tissue and 9 cases from excision specimens (Fig. 1). The number of interpretable individual ductal profiles within each clinical case ranged from 2 to 24 with positive object counts (individual nuclei with detectable staining) ranging from 7 to 90, demonstrating a dynamic range of mean nuclear DAB from 2 to 240 (median 16.5).

### Carcinoma clinical control cases

Thirty-four control cases of invasive carcinoma stained for ER during the 7-year period were identified with internal normal background epithelium from 22 core biopsies and 12 excision specimens. Normal ER clinical control cases showed no significant difference from the patients with low ER positive tumors in patient age or tumor HER2 status (Table 1). Control cases were more likely to be of lower grade, and more diffusely ER and PR positive. The number of interpretable individual ductal profiles within each normal ER control case ranged from 2 to 30 with positive object counts (individual nuclei with detectable staining) ranging from 3 to 182, demonstrating a dynamic range of mean nuclear DAB from 3 to 475 (median 30.8).

**Table 1:** Clinicopathologic features of low ER positive and clinical control cases.

Variable	Low ER cases (*n* = 21)	Control cases (*n* = 34)	*P*-value
Mean (range) patient age	56 (33–75)	59(29–80)	0.506
Tumor grade			0.001
1 (well differentiated)	1	15	
2 (moderately differentiated)	8	13	
3 (poorly differentiated)	12	6	
Tumor mean (range) % ER positive staining	7.2 (1–10)	83 (0–100)	<0.001
Tumor mean (range) % PR positive staining	6 (0–90)	58 (0–100)	<0.001
Tumor Her2 status*			0.227
Negative	15	30	
Positive	6	4	

### Normal benign breast control cases

**Fig. 2:**
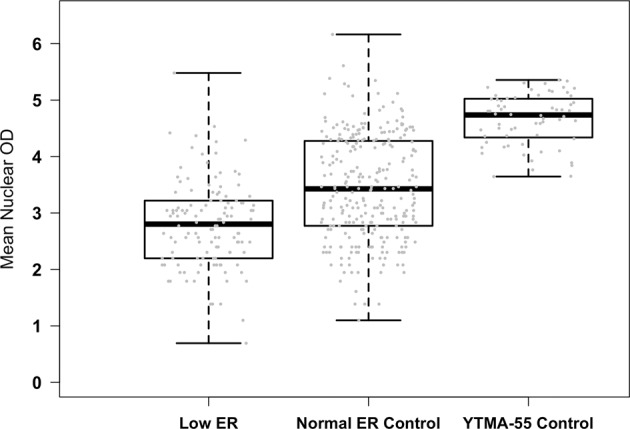
Mean Nuclear OD in Low ER and control groups. The mean nuclear OD values were log transformed. Cases of Low ER (1–10% staining) are compared to normal epithelium in the matched clinical full section control cases and in core tissue on YTMA-55. The lower and upper bars represent the minimum and maximum, respectively; the lower and upper edges of the box represent the 25th and 75th percentiles, respectively; the line in the middle of the box represents the median; the gray dots represent log transformed mean nuclear OD values.

Normal breast epithelium was present in 10 core samples from YTMA-55. The number of interpretable individual ductal profiles within each clinical case ranged from 2 to 10 with positive object counts (individual nuclei with detectable staining) ranging from 2 to 51, demonstrating a dynamic range of mean nuclear DAB from 38.3 to 212 (median 114). As expected, there was no correlation between the mean nuclear DAB OD and positive object count (*r* = 0.05, *p* = 0.29; Supplementary Fig. 1). Mean nuclear DAB of the low ER cases were significantly lower than that of the normal ER control cases (*p* < 0.001) and the YTMA-55 control cases (*p* < 0.001) based on linear mixed effects model (Fig. 2).

## Discussion

The hormone receptor and HER2 status of breast carcinomas is the foundation on which treating physicians determine appropriate clinical management for patients. Since multiple pre-analytic, analytic and interpretive variables can influence the results of these tests that are ultimately reported, this testing is highly regulated. Although most breast carcinomas are distinctly ER positive or negative,^11^ a small subset of tumors (between 1 and 5%) have been repeatedly recognized as demonstrating a low level of ER that may be troublesome from interpretive and treatment perspectives.^11,15^ While it may be unclear if this patient subset will benefit clinically from endocrine therapy, the pathologist must first ensure the testing was performed properly. In this study we utilized digital quantification to accurately measure the range of ER expression in normal background epithelium as internal control tissue to assess adequate ER staining of tumor cells by IHC. We found that internal control epithelium expresses a lower dynamic range in sections with tumors demonstrating low ER expression than was seen in the normal epithelium of control tissue, suggesting an inadequacy of the staining process in these particular clinical cases.

The cases in our study were selected based on a primary pathologist interpretation and reporting of low ER (1–10%) expression. This specific level of ER expression has been previously shown to be a category of tumors with low interobserver reproducibility for visual ER quantification by breast pathologists.^15^ Studies have found that most breast carcinomas express ER in a bimodal distribution, being completely negative or diffusely positive in up to 99% of cases, reflecting their typically monoclonal biology,^11^ with one study going so far as to suggest quantification of ER is therefore unnecessary.^13^ In these studies, however, ER expression was visually estimated by pathologists, potentially reflecting a human inability to recognize the full range of biologic ER expression in tumor cells. Rimm et al compared pathologist interpretation of ER IHC staining to quantitative assessment by automated digital analysis with the Aperio nuclear algorithm, demonstrating that visual interpretation did indeed produce a bimodal distribution compared to the more continuous range of expression seen by automated quantitative measures.^18^ As such, it was important to use digital analysis in our study to more precisely evaluate the full range of ER expression in normal epithelium.

The degree of ER expression in normal breast epithelium has been shown to be dependent on biologic factors such as menopausal status and body mass index but may also be related to breast cancer risk and affected by timing within the menstrual cycle and the presence of an adjacent breast carcinoma.^19–23^ It has been theorized that ER positive tumors may cause a “field effect”, resulting in higher ER expression in normal breast epithelium adjacent to a strongly ER positive tumor due to increased ER gene expression in normal terminal ductal lobular units of patients with ER positive breast carcinomas.^24^ However, results of studies evaluating protein expression of ER by IHC in normal epithelium are conflicting. Yang et al. used image analysis to compare ER expression in epithelium located near breast carcinomas to the expression levels in terminal ductal lobular units located distant from the tumor based on TMA sections.^23^ They found significantly higher levels of ER expression near the tumors but the level of expression did not correlate with ER expression in the carcinoma cells, arguing against a positive field effect by the tumor. Increased ER expression in normal breast epithelium has been consistently shown to be an independent risk factor for breast carcinomas and increased expression in epithelium near a primary tumor may instead represent one manifestation of early malignant change.^20–22^ An additional study utilizing digital analysis of TMA sections found higher ER expression in background epithelium of patients with ER negative and triple negative tumors, further arguing that increased ER expression represents a risk marker rather than a result of field effect from adjacent tumor.^19^ Conversely, Khan et al. found no relation of ER expression to the presence of carcinoma but this was a much smaller patient sample size, utilizing full tissue sections.^21^ In our current study, background ER expression in both the low ER cases and the full section control cases were assessed in benign ducts in the same tissue sections as the invasive carcinoma, negating the potential of expression differences due to distance from tumor.

The definition of ER positivity of at least 1% ER staining at any intensity by IHC has been debated in the literature but remains the clinically validated cutoff at which clinical benefit from endocrine therapy has been shown.^9^ Although studies have shown high concordance between ER expression by RNA and IHC, they suggest that the 1% IHC threshold may not correspond perfectly with a positive ER result by RNA. In one study assessing mRNA levels by TargetPrint, most breast carcinomas (five of eight cases) with 1–10% ER expression by IHC were negative by RNA analysis.^25^ In this study however, there were even more cases (*n* = 15) with negative IHC ER results that were ER positive by RNA analysis. Similarly, an additional study found a subset of cases interpreted as ER negative by IHC but positive by RNA expression using the GeneChip Human Genome U133 Plus 2.0 array.^26^ When utilizing Onco*type* DX RNA quantification, IHC ER-negative cases that were RT-PCR positive were more common than IHC ER-positive cases that were RT-PCR negative, suggesting IHC finding may under-represent true ER expression at the RNA level in a subset of cases.^27^ These studies show the challenges of definition of a precise biological cut point near the limits of detection for ER.

There are some inherent limitations in a study such as this. Many variables influence the level of ER expression in benign breast epithelium and some of these factors are unknown in our patient and control cases. The TMA cases were collected from deidentified breast cases and it is therefore not possible to determine any biologic factors that may have influenced normal ER expression. The TMA controls were stained with the same antibody but under a slightly modified protocol and in this study act as control for the multiple potential biologic effects on ER expression. Conversely, the full section control cases in our study were stained under the same protocol as the reported low-ER cases, thereby controlling for possible analytic variations that may have occurred from week to week within the clinical laboratory during the staining process over the 7-year period. The patient characteristics for cases within the low ER and control subsets are not all equally matched as evidenced by significant differences in tumor grade and ER status (Table 1). The difference in ER status in these cases cannot be matched as, by study design, we are focusing on a specific subset of low-ER tumors. Lower or negative ER expression is seen more frequently in higher grade tumors, reflecting the difference in tumor grade between the patient groups. Nevertheless, ER expression in normal epithelium has not been shown to vary with tumor grade.^19^ Tissue samples for our TMA control cases were collected more than 30 years ago and passage of time has been shown to reduce antigenicity of formalin-fixed paraffin-embedded tissue.^28^ If there was any loss of antigenicity in these cases however, it would only further strengthen our findings, as the TMA control cases still showed higher ER expression overall than was seen in normal epithelium of low ER positive cases. An additional limitation is the small number of cases in our study with low ER expression, a number that was further reduced by the absence of normal internal control epithelium in some cases. This highlights the expected rarity of these cases in daily practice and is supported by prior studies showing most breast carcinomas demonstrating diffuse or negative ER expression.^11,13^ Of the cases studied here, only two cases showing low ER expression on core biopsy underwent repeat staining on the excision specimen and remained in the 1–10% expression range. Scientific testing to determine if repeat testing would alter the ER expression, as hypothesized in this study, would require repeat testing on a larger number of low ER tumors and performance of repeat testing on the same specimen as was originally stained, in the same stainer run. Moreover, the comparison of core biopsy ER results to the ER results on the subsequent excision specimen introduces the confounding factors of tumor heterogeneity and pre-analytic variation. These two cases do not contribute statistically significant data to disprove our hypothesis.

In summary, we have shown that the dynamic range of ER expression in the normal background epithelium surrounding breast carcinomas with low (1–10%) ER expression is lower than that of normal epithelium in other patient tissue samples, suggesting a weakness in the staining process rather than a reduction in biologic expression of ER in those tumors. Assessment of ER expression in normal internal control tissue, when present, is necessary but the interpreting pathologist cannot accurately visualize the dynamic range of the staining. Digital analysis of these cases would be required to analyze the dynamic range as a control for staining quality but this would be a time-consuming and impractical solution for most labs. We would therefore suggest repeat ER IHC testing of the specimen in cases with low ER tumor expression to ensure adequate staining, even when “positive” staining of normal internal control epithelium is seen.

## Methods

### Clinical case selection and controls

A retrospective pathology report search of the Yale Pathology electronic pathology record was performed to identify invasive breast carcinomas with reported low ER expression (defined as 1–10% expression of any intensity) between the years of 2011 and 2018, and interpreted using 2010 ASCO/CAP criteria.^6^ Glass slides of the original ER stained sections were retrieved from the archive for digital scanning. Pathologic and clinical features including patient age at the time of diagnosis, tumor grade, ER, PR and HER2 status were obtained from the pathologic report (Fig. 1).

We used two different normal controls. The first control set utilized a tissue microarray (TMA), YTMA-55, constructed of tissue obtained from the archives of the Pathology Department at Yale University, consisting of 0.6 mm cores of normal breast tissue collected in 1981 and 1982. This set was utilized as a normal level of ER expression in benign breast tissue. The second group of normal control cases included benign background epithelium from sections of invasive breast carcinomas tested for ER by IHC in the Yale clinical laboratory. These cases were identified by a retrospective electronic pathology record search, limited to those with ER testing performed +/− 4 days from the date of a reported low (1–10%) ER clinical case. This second control set was utilized specifically to evaluate the staining of background epithelium in true clinical cases that were stained under the same pre-analytical and analytical conditions as were present when staining was performed on the low ER study cases. Tissue and associated clinico-pathological information were used after approval from the Yale Human Investigation Committee (protocol # 9505008219). Given the retrospective nature of the study, a waiver of written consent was granted.

### Immunohistochemistry

Automated IHC was performed on clinical patient and control cases: Slides were baked at 60 °C for 30 minutes. The Leica Bond III (Buffalo Grove, IL) was utilized to deparaffinize and perform high pH retrieval for 20 min at 95 °C. For chromogenic visualization, slides were incubated for 20 minutes with the rabbit monoclonal SP1 antibody (Cell Marque, Rocklin, CA, 1:50 dilution). Following proprietary post-primary application, a labeled polymer was applied for 8 minutes. An endogenous peroxidase block was applied for 5 minutes, followed by 3,3′-diaminobenzidine (DAB) application for 10 min. Slides were autostained with a 5 min hematoxylin counterstain application on the machine and 30 s off the machine. The slides were dehydrated through ethanol, cleared through xylene and coverslipped. This was the staining process utilized for all clinical cases throughout the 7-year study period. Staining was not repeated on clinical control or patient study cases.

Manual IHC was performed on YTMA-55: Slides were baked at 60 °C for 30 min to remove excess paraffin. Deparaffinization was performed in xylenes for two periods of 20 min each, after which slides were transferred to 100% ethanol and rehydrated to water in grades of ethanol. Heat-induced antigen retrieval took place in a PT module (LabVision, Kalamazoo, MI), where slides were immersed in sodium citrate buffer (pH6) for 20 min at 97 °C. Slides were then rinsed in distilled water, transferred to a solution of 0.75% H_2_O_2_ in methanol for 30 min at room temperature to block endogenous peroxidases, and rinsed again in distilled water. They were then transferred to a Labvision autostainer, where the remaining staining steps were performed at room temperature and rinsed with tris-buffered saline/0.05% Tween-20 (TBST) between each stage. Nonspecific antigens were blocked by 30 min in 0.3% bovine serum albumin (BSA) diluted in TBST. For chromogenic visualization, slides were incubated for 1 h with the rabbit monoclonal SP1 antibody (ThermoScientific, Waltham, MA, 1:100 dilution) in BSA-TBST, then anti-rabbit EnVision (Dako) for 1 h. Signal was developed for 5 min in DAB solution (Dako; prepared according to manufacturer instructions), followed by counterstaining for 1 min with hematoxylin (Tacha’s automated hematoxylin, BioCare Medical, Concord, CA). Slides were removed from autostainer and coverslipped.

### Quantitation of IHC

**Fig. 3:**
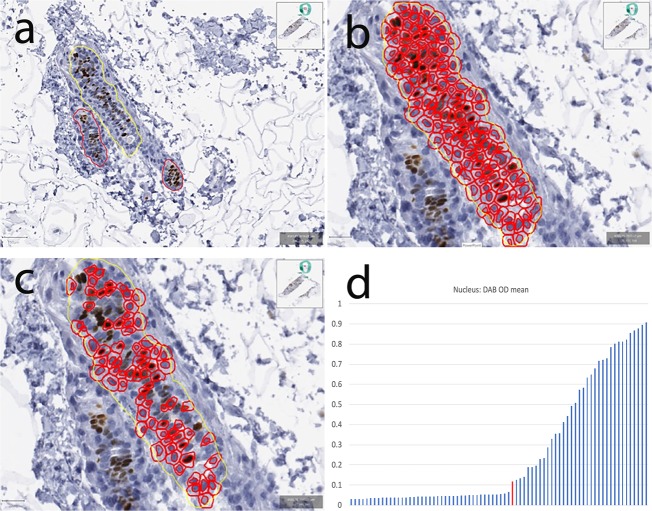
Cell detection and ER quantification on normal ducts using the open-source platform QuPath. **a** Non-neoplastic breast duct epithelium manually demarcated on a whole-slide image, outlined in yellow. **b** Initial detection of luminal nuclei circled in red. **c** Following manual elimination of objects deemed to represent non-luminal nuclei or incorrect partitioning of luminal nuclei. **d** Dynamic range of ER expression plotted with each bar representing the mean DAB for individual nuclei (*100) and the red bar representing the lowest limit of detection. OD = optical density.

Whole-slide images were scanned using Aperio ScanScope CS (Aperio Technologies, Vista, CA). Digital slide images were then imported into QuPath, Version 0.1.2, an open-source pathology software platform.^29^ Within QuPath, red-green-blue (RGB) color vector estimation was performed using the auto detect feature of the visual stain editor as preprocessing for every whole-slide image. Using QuPath, normal, non-neoplastic breast duct epithelium was manually demarcated on whole-slide images and verified by a pathologist (Fig. 3a). For each region of normal breast epithelium, watershed cell detection parameters were established (Fig. 3b).

Detected objects for which any of the following was noted were not included in ER quantification: (1) incomplete nuclear separation (e.g., mean nuclear DAB value assessed as a function of color deconvolution within an area greater than or equal to two nuclei); (2) incorrect partitioning of the nucleus–cell separation incompatible with nuclear morphology (e.g., mean nuclear DAB value assessed as a function of color deconvolution within an area less than one nuclei); (3) identification of an object which did not correspond to a nucleus (Fig. 3c).

ER staining for each cell was quantified as the mean optical density (OD) of nuclear DAB staining. The dynamic range for each outlined non-neoplastic duct was calculated as nuclear DAB OD mean: ((highest+) − (lowest+)) * 100 (Fig. 3d).

### Statistical analysis

The Student’s *t*-test was used to compare continuous variables, whereas *Fisher’s exact test* was used to compare categorical variables. Spearman’s rank correlation coefficient (*r*) was used to measure the association between two continuous variables. To account for the correlation of data points from the same patient, linear mixed effects model was used to assess the difference between the low ER group and each of the control group. Mean nuclear OD values were log transformed to maintain the normality assumption. As the accuracy of mean nuclear OD values might depend on the number of positive cell counts, we also modeled the variance of mean nuclear OD, allowing it to change proportionally to a power of positive cell counts. The variance structure was modeled in several ways and the choice of variance function was based on likelihood ratio test and Akaike’s information criterion. Two-sided P values of less than 0.05 were considered to indicate statistical significance. Analyses were performed with the use of R version 3.5^30^ and R package nlme.^31^

### Reporting summary

Further information on research design is available in the Nature Research Reporting Summary linked to this article.

## Supplementary information

Supplemental Figure 1

Reporting Summary Checklist

## Data Availability

The data generated during the current study (Supporting Figs. 2 and 3) are publicly available in the figshare repository: 10.6084/m9.figshare.11482665. The data supporting Table 1 of the published article are not publicly available in order to protect patient privacy, but can be made available on reasonable request from the corresponding author as described in the figshare record above.^32^

## Code availability

The open source software, QuPath, Version: 0.1.2, was utilized in data collection and analysis performed using R version 3.5 as described and reference in the method section.
