# Supplementary material for: Association between low estrogen receptor positive breast cancer and staining performance
Source: NPJ Breast Cancer. 2020 Feb 5;6:5. doi: 10.1038/s41523-020-0146-2 (PMC7002746; doi:10.1038/s41523-020-0146-2)

Supplementary Figure 1: Mean Nuclear OD and positive object count from all patients. The mean nuclear OD values were log transformed. Mean nuclear OD was not associated with positive object count (r=0.05, p=0.29).

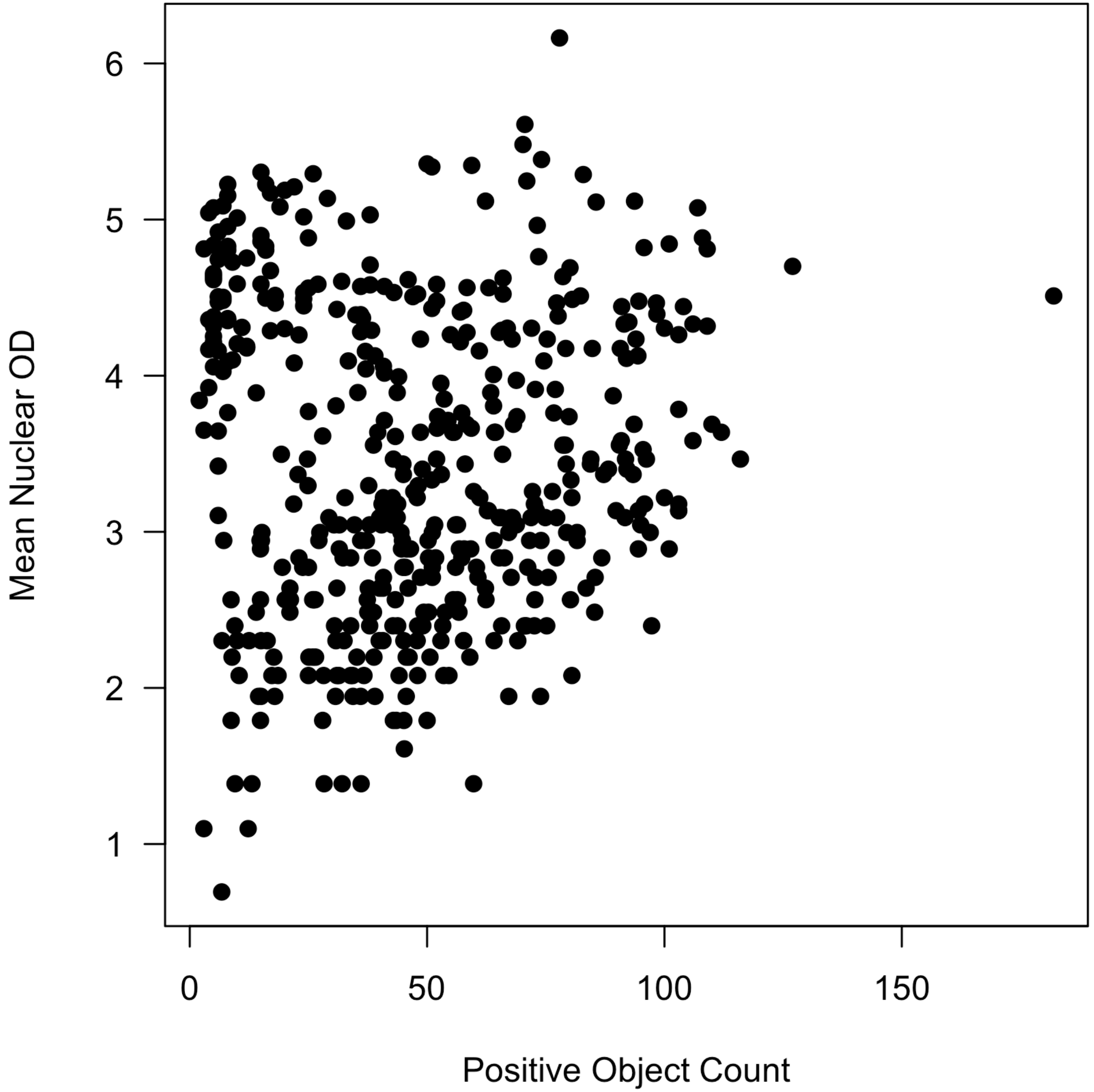

Supplement: Supplementary file 1 — Supplemental Figure 1 [file 41523_2020_146_MOESM1_ESM.pdf]
